# A Novel Technique to Reduce Reliance on Opioids for Analgesia from Acute Appendicitis: The Ultrasound-guided Erector Spinae Plane Block

**DOI:** 10.5811/cpcem.2019.4.42117

**Published:** 2019-05-29

**Authors:** Daniel Mantuani, Josh Luftig, Andrew Herring, Andrea Dreyfuss, Arun Nagdev

**Affiliations:** Highland General Hospital, Alameda Health System, Department of Emergency Medicine, Oakland, California

## Abstract

Single injection, ultrasound-guided nerve blocks have drastically changed the multimodal approach to pain management of the acutely injured patient in the emergency department (ED). Ultrasound-guided femoral nerve blocks in the ED have become standard aspects of multiple, hospital system pain management protocols, with early evidence demonstrating improved patient outcomes. Developing a multimodal pain management strategy can improve analgesia while reducing reliance on opioids in this era of opioid addiction.^1^ The single injection, ultrasound-guided erector spinae plane (ESP) block is a technique safely used for pain control for rib fractures that can be easily performed at the bedside and integrated into optimal emergency care. A more inferiorly located ultrasound-guided ESP block has been recently described in the anesthesia literature for perioperative pain control for various abdominal surgeries but has not yet been described for patients with acute appendicitis. Here we describe a single injection, lower ESP block performed by emergency physicians that successfully alleviated pain from acute appendicitis in an ED patient awaiting definitive surgical treatment. Along with allowing clinicians to actively manage pain without reliance on opioids, this novel ED technique may improve patient care outcomes.

## INTRODUCTION

Acute appendicitis is commonly diagnosed in the emergency department (ED), where intravenous (IV) opioids are the primary analgesic used to control pain while patients await definitive surgical care.^2^ Increasingly, data support the benefits of opioid-sparing multimodal analgesia for intra-abdominal surgery as part of enhanced recovery after surgery protocols.^3^ However, little has been studied in the preoperative period, specifically while patients are in the ED. Ultrasound-guided nerve blocks have become standard practice for many emergency physicians (EP) for acute traumatic injury such as hip fracture. The ultrasound-guided erector spinae plane (ESP) block, developed for both chronic and surgical thoracic pain and later adopted by EPs for management of acute rib fractures, is thought to provide relief for both somatic and visceral pain.^4^-^6^

Recently, several reports suggest benefit of the ESP block for the pain associated with major open abdominal surgery, bariatric surgery, ventral hernia repair, abdominal zoster, laparoscopic cholecystectomy, inguinal hernia repair, and cesarean section.^6^-^11^ This single injection, interfascial plane block could be an ideal pain-control intervention for the ED patient awaiting definitive surgical intervention. Along with offering effective localized pain management, the ESP block could potentially reduce or even obviate the need for opioids for acute appendicitis. Herein, we present the first description of a successful, ultrasound-guided ESP block for pain control in preoperative acute appendicitis.

## CASE REPORT

A 24-year-old male presented to the ED after seven hours of sharp mid-abdominal pain migrating to the right lower quadrant (RLQ), associated with nausea and vomiting. Vital signs were within normal limits except for mild hypertension (149/68 millimeters of mercury). Laboratory results showed a leukocytosis, white blood cells 14.5 x 10^9^/liters (L) (4.5-11.5 x 10^9^/L). A contrast-enhanced computed tomography of the abdomen and pelvis revealed a 10-millimeter (mm) dilated, fluid-filled appendix with appendicolith suggestive of acute appendicitis. The patient was given IV cefoxitin and admitted to the department of surgery for planned appendectomy later that day. For analgesia, the patient received 0.5 milligrams (mg) IV hydromorphone, 30 mg IV ketorolac, and 1000 mg IV acetaminophen but was still reporting 7/10 pain. Palpation of the RLQ produced 10/10 sharp localized pain with rebound and guarding. Operative management was to be delayed for four hours; thus, after discussion with the surgical team, the patient was offered a single injection, ultrasound-guided ESP (block) for pain control.

**Image 1:**
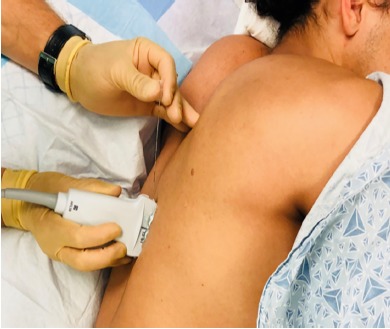
In preparation for administration of erector spinae plane block, the patient is positioned in either lateral decubitus with the affected side up or prone with the provider at the head of the bed. The needle trajectory is from cranial to caudal.

**Image 2:**
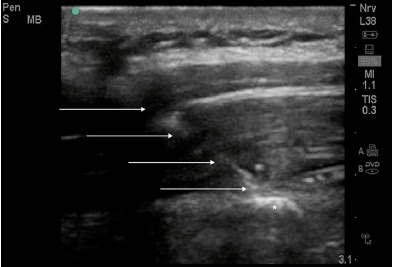
In-plane approach: advancing the needle tip (arrows) through the erector spinae muscle to the posterior surface of the transverse process of the first lumbar vertebra (*).

After the patient gave verbal consent he was placed on a cardiac monitor and rolled into the left lateral decubitus position. The physician stood at head of the bed facing the patient’s feet with the ultrasound monitor in direct line of sight (Image 1). The first lumbar (L1) spinous process was identified using surface landmarks and ultrasound imaging and the skin cleansed with chlorhexidine. A high-frequency 10-5 megahertz linear ultrasound probe was placed in the parasagittal plane approximately three centimeters (cm) lateral to the spinous process at the right L1 transverse process. Under ultrasound-guidance, a 20 gram (g)-3.5 inch Touhy needle was advanced in-plane, cranial to caudal, until the needle tip was in contact with the posterior surface of the transverse process (Image 2). Hydrodissection with 10 milliliters (mL) of normal saline with direct visualization confirmed needle tip placement in the fascial plane adjacent to the posterior surface of the transverse process. After negative aspiration, 20 mL of 1% lidocaine with epinephrine was injected in aliquots of 2-4 mL.

**CPC-EM Capsule**

What do we already know about this clinical entity?

Traditional pain control for acute appendicitis relies on intravenous opiates.

What makes this presentation of disease reportable?

Regional anesthesia has not been described for analgesia in acute appendicitis.

What is the major learning point?

A single injection, ultrasound-guided erector spinae plane block can provide complete analgesia for appendicitis.

How might this improve emergency medicine practice?

Emergency physicians can now incorporate regional anesthesia for pain control in acute appendicitis.

Thirty minutes after the procedure the patient reported 0/10 pain at rest. With deep palpation to the RLQ, the patient reported mild, dull, pressure-like pain (3/10); the abdomen remained soft without guarding, rebound or localized pain. Sensory testing to cold with ethyl chloride spray revealed right-sided decreased sensation in the tenth thoracic (T10) to L2 distribution (from approximately two cm inferior to the umbilicus to the mid-anterior right thigh). Two hours later, nursing documentation indicated that the patient’s pain remained well controlled. During the remaining 5.5 hours of his ED stay the patient reported his pain was well controlled and required no additional analgesic medications. He had an uncomplicated laparoscopic appendectomy, with appendicitis later confirmed by pathology.

## DISCUSSION

Currently, pain control for acute surgical abdominal pain is often limited to IV opioids, sometimes combined with nonsteroidal anti-inflammatory drugs or acetaminophen. With operating room crowding, abdominal surgical patients often board in the ED for many hours, relying on repeated dosing of opioid medications that risk side effects while often failing to provide adequate pain relief. In the last quarter of 2018 at our institution, patients admitted for appendicitis boarded an average of 320 minutes before leaving the ED. Ultrasound-guided regional blocks for abdominal surgeries performed by anesthesiologists have been shown to be effective in the perioperative and postoperative period.^8^,^10^,^11^ This is the first report of an ESP block for acute appendicitis performed by EPs on a patient boarding in the ED whose pain was unrelieved by IV hydromorphone, acetaminophen, and ketorolac. This technique can be incorporated into an opioid-sparing, multimodal pain control strategy for patients with confirmed appendicitis who are boarding in the ED, and it may apply to other painful surgical abdominal pathologies.

Communication with surgical consultants with regard to performing the ESP block before the procedure should be routine. Clear documentation in both the chart and on the patient (we commonly place the time and block type) should also be part of the workflow when incorporating ultrasound-guided nerve blocks in the ED. Surgical consultants should also be aware that peritoneal blocks may significantly reduce the pain from acute appendicitis; thus, using the abdominal examination as a marker of progression of illness may not be possible.

Technical considerations include adhering to block safety standards to prevent local anesthetic systemic toxicity. Because this was a novel, lower-targeted ESP block, we used a shorter acting anesthetic (1% lidocaine with epinephrine) as an additional precaution in case of adverse effects. However, a longer-acting anesthetic (such as bupivacaine or ropivacaine) would likely be optimal and consistent with prior published reports of ESP block.^13^ In the thorax, injecting into the erector spinae fascial plane spreads multiple vertebral levels superiorly and inferiorly.^5^,^6^ Additional study is needed to determine the ideal vertebral level needed to cover the lower abdomen. Previous case reports in the anesthesiology literature indicate that ESP block placed at the T8-T9 level may be preferred as it is less likely to cause excessive lower lumbar blockade, and it is easier to target since the transverse process is located more superficially at that level.^7^-^9^,^11^,^12^

## CONCLUSION

Given our experience managing pain for rib fractures, we consider the ESP block to be technically feasible and highly effective. By moving the location of the single injection ESP block more caudally, we can expand the range of this block to include pain control for acute appendicitis as part of an opioid-sparing, multimodal pain control strategy for patients in the ED. We suspect that further study of this novel technique may show improved pain control for patients with acute appendicitis when compared to standard opioid-based pain regimens with fewer side effects and improved patient outcomes.
